# Association of Influenza Vaccination and Prognosis in Patients Testing Positive to SARS-COV-2 Swab Test: A Large-Scale Italian Multi-Database Cohort Study

**DOI:** 10.3390/vaccines9070716

**Published:** 2021-07-01

**Authors:** Marco Massari, Stefania Spila-Alegiani, Massimo Fabiani, Valeria Belleudi, Gianluca Trifirò, Ursula Kirchmayer, Francesca Romana Poggi, Pamela Mancuso, Francesca Menniti-Ippolito, Rosa Gini, Claudia Bartolini, Olivia Leoni, Michele Ercolanoni, Filippo Da-Re, Stefano Guzzinati, Nicoletta Luxi, Flavia Riccardo, Paolo Giorgi-Rossi

**Affiliations:** 1 Pharmacoepidemiology Unit, National Centre for Drug Research and Evaluation (CNRVF), Istituto Superiore di Sanità, Viale Regina Elena 299, 00161 Rome, Italy; marco.massari@iss.it (M.M.); francesca.menniti@iss.it (F.M.-I.); 2 Department of Infectious Diseases (DMI), Istituto Superiore di Sanità, Viale Regina Elena 299, 00161 Rome, Italy; massimo.fabiani@iss.it (M.F.); flavia.riccardo@iss.it (F.R.); 3 Department of Epidemiology ASL Roma 1, Lazio Regional Health Service, Via Cristoforo Colombo 112, 00147 Rome, Italy; v.belleudi@deplazio.it (V.B.); u.kirchmayer@deplazio.it (U.K.); f.poggi@deplazio.it (F.R.P.); 4 Department of Diagnostics and Public Health, University of Verona, Piazzale L.A. Scuro 3, 37134 Verona, Italy; gianluca.trifiro@univr.it; 5 Italian Society of Pharmacology, Via Giovanni Pascoli 3, 20129 Milan, Italy; 6 Epidemiology Unit, Azienda Unità Sanitaria Locale-IRCCS di Reggio Emilia, Via Giovanni Amendola 2, 42122 Reggio Emilia, Italy; pamela.mancuso@ausl.re.it (P.M.); paolo.giorgirossi@ausl.re.it (P.G.-R.); 7 Agenzia Regionale di Sanità della Toscana, Via Pietro Dazzi 1, 50141 Florence, Italy; rosa.gini@ars.toscana.it (R.G.); claudia.bartolini@ars.toscana.it (C.B.); 8 Department of Health of Lombardy Region, Epidemiology Observatory, Piazza Città di Lombardia 1, 20124 Milan, Italy; Olivia_Leoni@regione.lombardia.it (O.L.); michele.ercolanoni@ariaspa.it (M.E.); 9 Regional Directorate of Prevention, Food Safety, Veterinary Public Health, Regione del Veneto, Rio Novo-Dorsoduro 3493, 30123 Venice, Italy; filippo.dare@regione.veneto.it; 10 Azienda Zero, Regione del Veneto, Passaggio Luigi Gaudenzio, 1, 35131 Padova, Italy; stefano.guzzinati@azero.veneto.it; 11 Department BIOMORF, University of Messina, Piazza Pugliatti 1, 98122 Messina, Italy; nicoletta.luxi@unime.it

**Keywords:** influenza vaccine, COVID-19, prognosis, health database, Italy

## Abstract

To investigate the association of the 2019–2020 influenza vaccine with prognosis of patients positive for SARS-CoV-2A, a large multi-database cohort study was conducted in four Italian regions (i.e., Lazio, Lombardy, Veneto, and Tuscany) and the Reggio Emilia province (Emilia-Romagna). More than 21 million adults were residing in the study area (42% of the population). We included 115,945 COVID-19 cases diagnosed during the first wave of the pandemic (February–May, 2020); 34.6% of these had been vaccinated against influenza. Three outcomes were considered: hospitalization, death, and intensive care unit (ICU) admission/death. The adjusted relative risk (RR) of being hospitalized in the vaccinated group when compared with the non-vaccinated group was 0.87 (95% CI: 0.86–0.88). This reduction in risk was not confirmed for death (RR = 1.04; 95% CI: 1.01–1.06), or for the combined outcome of ICU admission or death. In conclusion, our study, conducted on the vast majority of the population during the first wave of the pandemic in Italy, showed a 13% statistically significant reduction in the risk of hospitalization in some geographical areas and in the younger population. No impact of seasonal influenza vaccination on COVID-19 prognosis in terms of death and death or ICU admission was estimated.

## 1. Introduction

The recently discovered severe acute respiratory syndrome coronavirus (SARS-CoV-2) can cause the coronavirus disease (COVID-19) [1]. COVID-19 was first reported in the city of Wuhan (China) on 31 December 2019 and has rapidly spread around the world [2]. As of week 2021-8, almost 21.8 million confirmed COVID-19 cases have been reported in the EU/EEA, with 531,869 deaths [3]. In the period preceding the marketing of the first specific COVID-19 vaccines, occurring in late December 2020 in Europe, it has been hypothesized that some existing vaccines that prevent other respiratory infections, such as the influenza vaccine, may play a role in COVID-19 through different mechanisms by reducing the risk of infection or improving the prognosis once infected [4,5,6].

Accordingly, evidence suggests that influenza virus infection may increase susceptibility to SARS-CoV-2 infection, letting the penetration of this coronavirus into the lungs through an upregulation of the angiotensin-converting enzyme 2 (ACE2) receptors [7].

So far, a few observational studies explored the association between influenza vaccination during the previous campaign and COVID-19 (Table S1) [5,8,9,10,11,12,13,14].

All these studies seem to suggest that influenza vaccine administration is associated with lower susceptibility to SARS-COV-2 infection, while controversial results on its effect on COVID-19 prognosis has been reported.

In addition, the influenza vaccine may have indirect effects on the COVID-19 pandemic by preventing respiratory co-infection due to influenza, facilitating differential diagnosis in patients with symptoms related to respiratory infections and overall reducing the burden of viral pneumonia on the healthcare system [6]. Altogether, these findings further support the current influenza vaccination campaigns, especially in frail patients.

The aim of this large-scale Italian multi-database cohort study is to investigate the association of the influenza vaccine during the period 2019–2020 relating to the prognosis of patients positive to nasal/oropharyngeal swab testing for SARS-CoV-2 in the first COVID-19 pandemic wave.

## 2. Materials and Methods

We described the methods and presented findings according to the reporting guidelines for observational studies based on routinely collected health data (The RECORD statement—checklist of items extended from the STROBE statement) (Table S1) [15].

### 2.1. Setting and Study Design

The study was conducted in four Italian regions (i.e., Lazio, Lombardy, Veneto, and Tuscany) and the Reggio Emilia province (Emilia-Romagna region). Overall, more than 21 million adults aged ≥ 18 years were residing on 1 January 2020 in the study area (42% of the country population in the same age group) [16]. According to data from the Italian integrated surveillance database, including information on all cases of reverse transcription-polymerase chain reaction (RT-PCR) SARS-CoV-2 infections collected by local public health departments, adult cases diagnosed in the study area accounted for 56% of all COVID-19 cases notified during the first phase of the pandemic in Italy, i.e., up to mid-May 2020 [17]. The first wave of the pandemic had different impacts between regions and between different provinces within regions, because of the mobility restriction and physical distancing measure. School closures in northern Italy from February 24 followed by the strict lockdown from March 11 were able to almost completely prevent the spread of infections in southern Italy, but were enacted too late for most of northern Italy, while central Italy had intermediate situations. Consequently, the pressure on both contact tracing and testing systems and hospitals was not even across Italy (Table 1).

We identified, from the local or regional COVID-19 integrated surveillance databases, the whole cohort of cases of SARS-CoV-2 infection laboratory-confirmed by RT-PCR (COVID-19 cases), aged ≥18 years and diagnosed from the beginning of the epidemic on 21 February 2020 to 17 May 2020. The cohort was followed until 16 June 2020, thus accounting for at least 30 days of follow-up.

The study was approved by the ethical committee of the Italian National Institute of Health (Prot. PRE BIO CE 01.001, 11 May 2020).

### 2.2. Data Sources

The information analyzed in this study was retrieved from several regional routinely collected healthcare databases: (1) COVID-19 surveillance database, including demographic characteristics of cases, date of diagnosis, and possible occurrence of COVID-19-related death, hospitalization, and intensive care unit (ICU) admission; (2) flu immunization registry, including information on date of administration and type of seasonal influenza vaccine; (3) data on prescribed and distributed drugs partially or completely reimbursed by the National Health Service (NHS), coded with the Anatomical Therapeutic Chemical (ATC) classification system; (4) hospital discharge database that, for all patients admitted to regional public and private hospitals, includes admission and discharge dates and up to six discharge diagnoses coded according to the International Classification of Disease, Clinical Modification, ninth version (ICD-9-CM); (5) co-payment exemption database, including underlying conditions and the release and expiry dates of cost exemptions granted for related health-care services (e.g., drugs prescriptions, laboratory exams, and medical visits).

**Figure 1:**
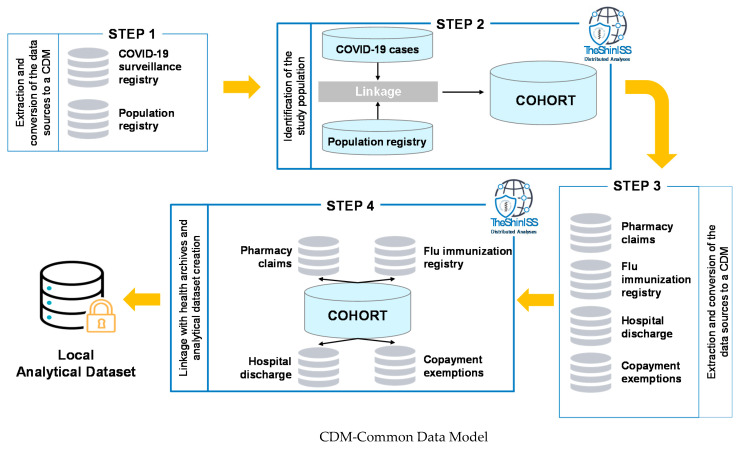
TheShinISS: an open-source tool for conducting distributed analyses within pharmacoepidemiological multi-database studies. Diagram of sequential steps applied to the cohort study design.

A free open-source statistical tool written with R language, “TheShinISS” [18], developed by the Italian National Institute of Health for the conduction of distributed analyses within the main epidemiological multi-database study designs, was delivered to the five study areas and locally used to perform data quality control and record linkage between the COVID-19 surveillance database and all the other local healthcare databases using an anonymized patient identification code; elaborating and processing health archives at local level; and finally creating the local anonymized dataset for the centralized data analyses (Figure 1).

### 2.3. Exposure, Outcomes, and Confounders

The exposure of interest was the administration of influenza vaccination in the season October 2019–January 2020.

The study outcomes were the occurrence of COVID-19 related hospitalization, death, and admission to ICU plus death (only limited to cases from Lazio, Lombardy, Veneto, and Tuscany, where information about admission to ICU was available) within 30 days from the date of SARS-CoV-2 infection, laboratory-confirmed by RT-PCR.

The following variables were considered as potential confounders: gender, age, geographic area, Charlson comorbidity index (CCI), based on hospitalizations in the previous ten years (categorized as 0, 1–2, and ≥3) [19], number of hospital admissions for any cause in the previous two years (categorized as 0, 1, ≥2), total number of prescriptions for any drugs in the previous year, prior use (at least one prescription in the previous year) of drugs for peptic ulcer and gastro-oesophageal reflux disease (GORD), anticoagulants, platelet aggregation inhibitors, lipid-lowering drugs, antibiotics, anti- human immunodeficiency virus (HIV), anti-Parkinson drugs, antiepileptics, antipsychotics, antidepressants, antiarrhythmics, corticosteroid for systemic use, disease modifying anti-rheumatics drugs (DMARDs), and recent use (≥1 prescription in the previous three months) of non-steroidal anti-inflammatory drugs (NSAIDs), history of pneumonia, cerebrovascular disease, cardiovascular disease, and hepatopathy, diabetes, dementia, hypertension, chronic renal failure, cancer, and rheumatic diseases. A detailed description of the ATC codes, ICD-9-CM codes, and cost-exemption codes used to identify the above listed types of drugs, causes of hospital admissions, and chronic conditions is presented in Table S2.

### 2.4. Statistical Analysis

We described the demographic and clinical characteristics of the cohort by exposure to seasonal influenza vaccination using frequency with percentages and median plus interquartile range (IQR) for categorical and continuous variables, respectively.

We used Poisson regression models with robust variance estimation to calculate the vaccinated-to-unvaccinated crude and adjusted Relative Risks (RRs), with their corresponding 95% confidence intervals (95% CI), for study outcomes within 30 days from the date of diagnosis. All the pre-specified potential confounders were considered eligible to be included in the multivariate models following a forward stepwise procedure based on the Akaike’s Information Criterion (AIC) method. We also conducted a supportive analysis using the individual matching propensity score method for adjustment.

Finally, we performed sub-group analyses by gender, age group (i.e., <65 years and ≥65 years), study center, calendar period of diagnosis (i.e., 21 February–20 March, 21 March–18 Apri, and 19 April–17 May), CCI, and the presence of chronic medical conditions in patients aged <65 years (i.e., diabetes, cardiovascular diseases, chronic renal failure, cancer, hepatopathy, and HIV).

All tests were two-sided and statistical significance was set at *p* < 0.05. The analyses were performed using R version 4.0.2 [20].

## 3. Results

From the local COVID-19 surveillance databases, we identified 116,283 COVID-19 cases aged ≥18 years during the period 21 February–17 May 2020. We excluded 80 cases (0.07%) who underwent seasonal influenza vaccination after 31 January 2020, and we also excluded 258 cases (0.22%) with incorrect dates.

Thus, 115,945 COVID-19 cases were finally included in the study, with 34.6% of them being previously vaccinated against influenza. Lombardy accounted for 68% of the entire cohort. Vaccinated and non-vaccinated patients, stratified by age group (<65 and ≥65 years), were statistically different for almost all variables (Table 2). Three different outcomes were considered for the risk estimates: hospitalisation (n. 53,054; 45.8%), death (n. 18,410; 15.9%), and ICU admission plus death composite outcome (n. 19,604; 17.5%).

### 3.1. Risk of Hospitalisation

The adjusted relative risk (RR) of being hospitalized in the vaccinated group as compared with the non-vaccinated group was 0.87 (95% CI: 0.86–0.88), showing a statistically significant risk reduction associated with flu vaccination (Table 3). The RR is only slightly closer to 1 when adjustment was performed using the propensity score (adj RR = 0.94, 95% CI 0.93–0.96).

Multivariate Poisson regression model (stepwise forward based on Akaike’s Information Criterion) adjusted for the following eligible variables: age, gender, centre, n. of hospitalisations, Charlson index, number of prescriptions, antiacid drugs, anticoagulants, platelet aggregation, lipid modifying agents, antibiotics, anti HIV drugs, anti-parkinsonian drugs, antiepileptics, antipsychotics, antidepressants, antiarrhythmics, NSAIDS, corticosteroids, DMARDs, hypertension, cerebrovascular diseases, hepatopathy, diabetes, dementia, chronic kidney failure, chronic pulmonary disease, neoplasms, artery cardiac disease, rheumatic diseases.

**Figure 2:**
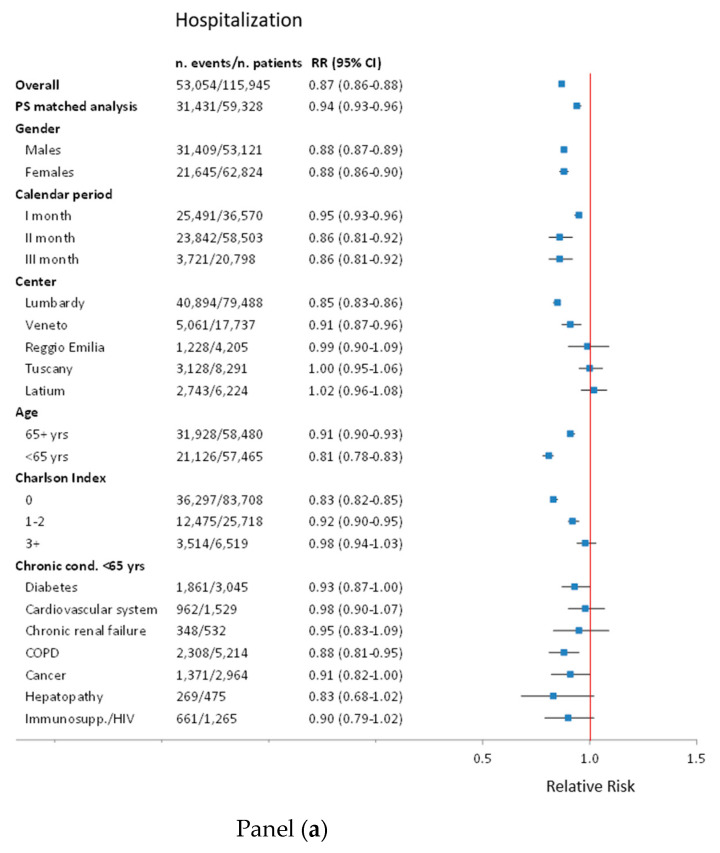

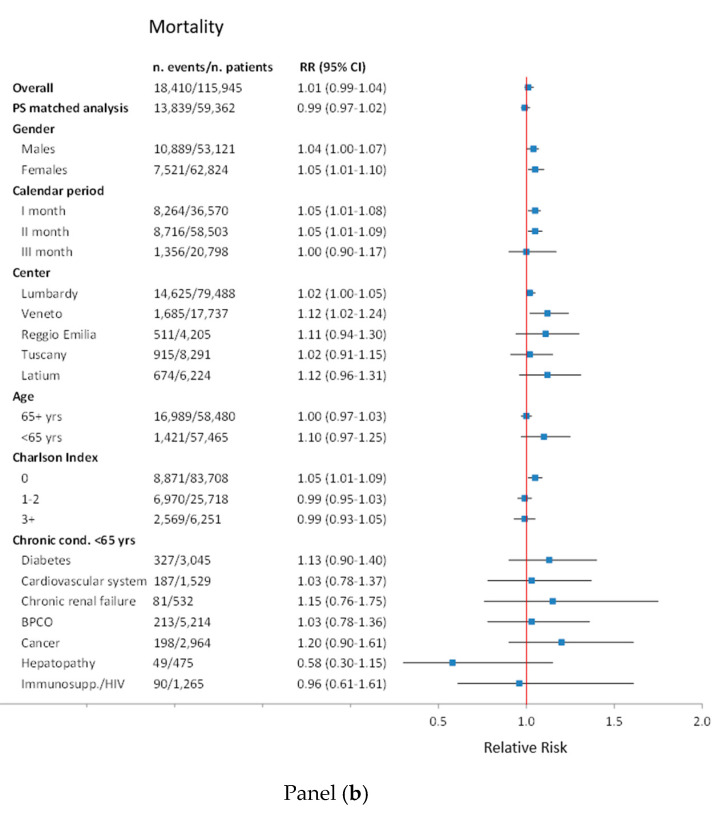

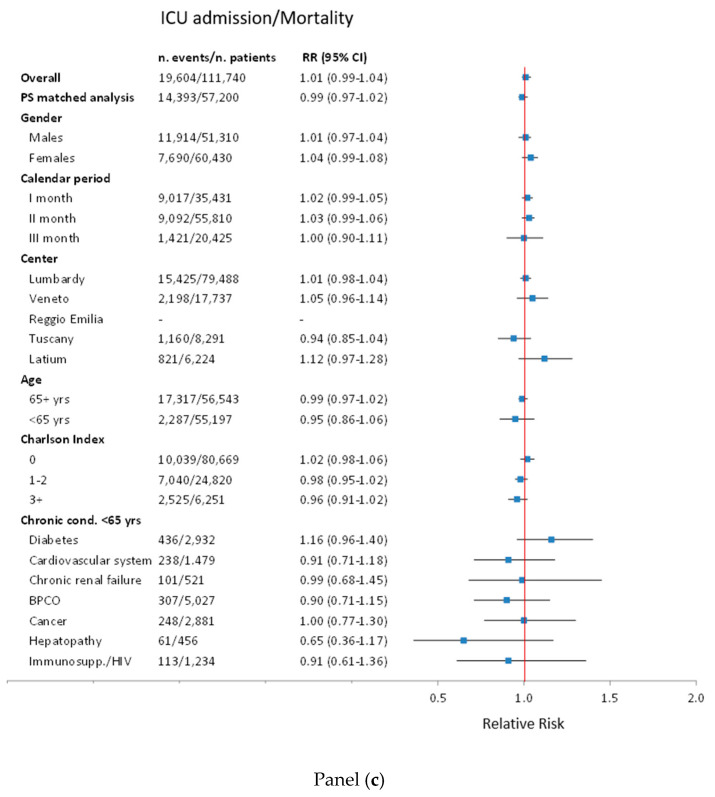
Sensitivity and subgroup analyses of association between flu vaccination and COVID-19 hospitalization panel (**a**), mortality panel (**b**), and admission to ICU or mortality panel (**c**). PS—Propensity Score; yrs—years; Cond.—Conditions; COPD—Chronic obstructive pulmonary disease; Immunos—Immunosuppression; HIV—Human Immunodeficiency Virus infection; ICU—Intensive Care Unit.

Subgroup analyses show that a risk reduction of COVID-19 related hospitalization was estimated mainly in Lombardy, less in Veneto, but not in Reggio Emilia, Tuscany and Latium. The effect was weaker in people over 65, where the reduction was less than 10%. A trend of the magnitude of reduction was observed for the three levels of the CCI, going from an RR of 0.83 (95% CI: 0.82–0.85) for CCI = 0, to 0.98 (95% CI: 0.94–1.03) for CCI ≥ 3. Chronic conditions in people aged below 65 show a similar reduction to that in all younger people (Figure 2, panel a).

### 3.2. Risk of Death and ICU Plus Death Composite Outcome

In the unadjusted analysis, vaccinated people presented a higher risk of death (RR = 2.36 95% CI: 2.29–2.42); however, the excess risk almost fully disappeared in age and sex adjusted analysis (adj. RR = 1.06; 95% CI: 1.03–1.09). Further adjustment for all the possible confounders only slightly modified the RR of risk of death (RR = 1.04; 95% CI: 1.01–1.06), while the adjusted RR for the combined outcome ICU admission or death was 1.01 (95% CI: 0.99–1.04) (Table 3). Results were similar among all sub-groups (Figure 2, panel b and c).

## 4. Discussion

This large cohort multi-regional Italian study showed no effect of vaccination on the hard outcome of death and on the universally accepted definition of very severe disease of ICU admission plus death composite outcome [21,22]. On the other hand, we observed a statistically significant reduction of COVID-19 related hospitalization in patients testing positive to SARS-COV-2 swab test who were previously vaccinated against influenza, which was appreciable in people below 65 years of age and without comorbidities.

Given the current guidelines for anti-influenza vaccination, prevalence of vaccination was higher in people over 65 and in people below 65 with comorbidities; in fact, vaccination is recommended for those under 65 years for many conditions that are known to be negative prognostic factors of COVID-19. Therefore, it is not surprising that crude RRs showed an excess risk of death in vaccinated people. Adjusted analyses for a large number of possible confounders, allowed by the high statistical power of our cohort, showed no excess risk of death alone, and death/ICU admission; however no protective effect for the most severe COVID19-related outcomes was suggested by our results. This finding is consistent with previous studies [13,23,24]. As in most of these studies, we cannot exclude a residual confounding of unregistered medical conditions associated with a worst prognosis in vaccinated people. This is also indicated by the decreasing trend of RRs in people with more comorbidities, where residual confounding is more unlikely [25]. Nevertheless, variations in RR were very small and all very close to one.

The Lombardy region, where the effect was most appreciable, contributed to 69% of the study population (COVID-19 cases) and drove the overall effect. However, this region did not share similar characteristics in terms of cumulative incidence testing strategies and case fatality rate, as shown in Table 1. This also suggests that the difference in the effect between geographical areas could not be attributed to testing capability or to the pressure on hospitals, but could be linked to hospitalization criteria or to anti-influenza vaccination policies, variables not included in the available databases.

It is worth noting that among younger people, vaccination is also recommended for health workers and people involved in essential public services; this group of vaccinated people has been likely to be more frequently exposed to infection and testing and, in the presence of relatively mild symptoms, they may also had a lower propensity to hospitalization, given their intrinsic autonomy ability in managing everyday activities; such a difference in mild cases could not interfere with outcomes in very severe cases.

In our study, we could only measure the effect on prognosis of the disease and not on the effect on infection itself, which could also be a plausible way of action of the influenza vaccine [6]. It has been suggested that the influenza, pneumococcal, and the tuberculosis Bacillus Calmette-Guérin (BCG) vaccines may have direct benefits concerning COVID-19 infection [5,26], but the underlying pre-clinical and clinical evidence has not yet been explored in detail. In particular, there is evidence that live attenuated vaccines, such as the BCG vaccine, seem to protect against other seemingly unrelated pathogens [4]. In addition, the role of other vaccines concerning more common respiratory diseases, such as influenza and Streptococcus pneumoniae-induced respiratory infections, also came under scrutiny because contracting these diseases may make persons more vulnerable to developing COVID-19 [6]. Besides direct effects, these vaccines may also have indirect benefits, such as reducing the burden of preventable super-infections among COVID-19 patients.

Finally, it could be speculated that the vaccination may reduce some relatively mild/moderate symptoms, thus reducing the need for hospitalization for younger and healthier patients, but not changing the prognosis in the fragile population.

## 5. Conclusions

In conclusion, our study, conducted on the vast majority of the population exposed to the first wave of the pandemic in Italy, showed no impact of seasonal influenza vaccination on COVID-19 prognosis in terms of either death or death plus ICU admission composite outcome. On the other hand, a marginal statistically significant reduction of COVID19-related hospitalization, mostly in younger and healthier population testing positive to the SARS-COV-2 swab test, was observed; thus, this requires additional investigation.

## Figures and Tables

**Table 1:** Demographic characteristics of the areas included in the study and main epidemiological parameters of the first wave of the COVID-19 pandemic, 21 February to 17 May 2020.

COVID-19 Cases (%)									
Region	Population ^a^ ≥18 yrs ^b^	Cumulative Incidence (Range between Provinces) ^c^	ICU ^d^ Occupation at the Peak ^c^	Positive Tests ^c^	Vaccination Coverage >64 yrs ^b^	Vaccination Coverage ≤64 yrs ^b^	Hospitalised	ICU ^d^	Case Fatality Rate (30 Days)
Lombardy	8,461,634	0.84 (0.38–1.76)	100.0	24.9	55.2	12.1	51.5	2.6	18.4
Veneto	4,128,295	0.39 (0.19–0.54)	43.2	6.9	63.0	11.7	28.5	4.3	9.5
Reggio Emilia	440,869	0.92	65.0 ^e^	27.7	59.9	14.2	29.2	-	12.2
Tuscany	3,169,250	0.27 (0.16–0.54)	64.1	6.7	54.6	13.2	37.7	4.3	11.0
Latium	4,933,338	0.13 (0.09–0.25)	24.0	4.5	60.1	12.4	44.1	4.0	10.8

^a^ ISTAT, 2019; ^b^ yrs—years; ^c^ Istituto Superiore di Sanità, May 14, 2020; ^d^ ICU—Intensive Care Unit; ^e^ Computed for the entire Emilia-Romagna Region because the management of ICU is regionalized.

**Table 2:** Demographic and clinical characteristics of COVID SARS-COV-2 positive patients by age group and previous flu vaccination.

	Age <65 Years			Age 65+ Years		
	Total Case (N.)	Vaccinated Cases (%)	*p*	Total Case (N.)	Vaccinated Cases (%)	*p*
**COVID-19 diagnosed cases**	57,465	7008 (12.2%)		58,480	33,094 (56.6%)	
**Centre**						
Lombardy	36,760	4428 (12.0%)	0.003	42,728	23,604 (55.2%)	<0.001
Veneto	10,059	1180 (11.7%)		7678	4835 (63.0%)	
Tuscany	4673	618 (13.2%)		3618	1980 (54.7%)	
Reggio Emilia	2268	322 (14.2%)		1937	1160 (59.9%)	
Latium	3705	460 (12.4%)		2519	1515 (60.1%)	
**Gender**						
Females	31,051	3845 (12.4%)	0.14	31,773	18,683 (58.8%)	<0.001
Males	26,414	3163 (12.0%)		26,707	14,411 (54.0%)	
**Age, year**						
18–49	27,358	2566 (9.4%)	<0.001			
50–64	30,107	4442 (14.8%)				
65–74				16,522	7125 (43.1%)	<0.001
75–84				21,349	12,400 (58.1%)	
85+				20,609	13,569 (65.8%)	
**Charlson index**						
0	52,225	5749 (11.0%)	<0.001	31,483	16,922 (53.7%)	<0.001
1–2	4786	1102 (23.0%)		20,932	12,479 (59.6%)	
≥3	454	157 (34.6%)		6065	3693 (60.9%)	
**N. of hospitalizations (last 2 years)**						
0	48,463	5512 (11.4%)	<0.001	38,185	21,505 (56.3%)	0.022
1	6375	946 (14.8%)		10,671	6167 (57.8%)	
≥2	2627	550 (20.9%)		9624	5422 (56.3%)	
**Comorbidities**						
Cerebrovascular diseases	900	250 (27.8%)	<0.001	9814	6016 (61.3%)	<0.001
Artery cardiac disease	1529	377 (24.7%)	<0.001	14,271	8731 (61.2%)	<0.001
Hypertension	12,841	2173 (16.9%)	<0.001	38,248	21,664 (56.6%)	0.73
Hepatopathy	475	110 (23.2%)	<0.001	1065	602 (56.5%)	0.97
Chronic kidney failure	532	152 (28.6%)	<0.001	3436	1972 (57.4%)	0.33
Diabetes mellitus	3045	699 (23.0%)	<0.001	12,078	7041 (58.3%)	<0.001
Chronic pulmonary disease	906	294 (32.5%)	<0.001	5908	3614 (61.2%)	<0.001
Neoplasms	2964	541 (18.3%)	<0.001	9081	5202 (57.3%)	0.15
Dementia	88	44 (50.0%)	<0.001	5117	3287 (64.2%)	<0.001
Rheumatic diseases	416	93 (22.4%)	<0.001	1029	604 (58.7%)	0.17
**Prior drug use ^a^**						
Antiacid drugs	9080	1551 (17.1%)	<0.001	23,474	13,385 (57.0%)	0.086
Lipid modifying agents	4791	1003 (20.9%)	<0.001	16,963	9633 (56.8%)	0.54
Anticoagulants	2962	484 (16.3%)	<0.001	11,402	6471 (56.8%)	0.70
Platelet aggregation inhibitors	2306	593 (25.7%)	<0.001	15,112	8824 (58.4%)	<0.001
Antiarrhythmics, class I and III	434	87 (20.0%)	<0.001	2927	1682 (57.5%)	0.33
Antibiotics	18,583	2485 (13.4%)	<0.001	21,226	11,927 (56.2%)	0.14
Anti HIV ^b^ drugs	316	74 (23.4%)	<0.001	264	128 (48.5%)	0.008
Anti-Parkinsonian drugs	116	40 (34.5%)	<0.001	1778	1099 (61.8%)	<0.001
Antiepileptics	2119	585 (27.6%)	<0.001	4857	2752 (56.7%)	0.92
Antipsychotics	1175	401 (34.1%)	<0.001	4621	2718 (58.8%)	0.001
Antidepressants	3794	667 (17.6%)	<0.001	9822	5840 (59.5%)	<0.001
Corticosteroids for systemic use	5347	845 (15.8%)	<0.001	7194	4024 (55.9%)	0.23
DMARDs ^c^	987	212 (21.5%)	<0.001	1183	602 (50.9%)	<0.001
**Recent drug use ^d^**						
NSAIDs ^e^	2234	329 (14.7%)	<0.001	3582	2006 (56.0%)	0.46

^a^ within the last available 12 months prior Index Date; ^b^ HIV—Human Immunodeficiency Virus; ^c^ DMARDs—disease-modifying antirheumatic drugs; ^d^ within the last available 3 months prior Index Date; ^e^ NSAIDs—non-steroidal anti-inflammatory drugs.

**Table 3:** Association between flu vaccination and COVID-19 related hospitalisation, mortality and admission to ICU plus mortality composite outcome.

	n. of Events/n. of Subjects		RR ^a^ unadj (95% CI ^b^)	RR adj (95% CI)
	Vaccinated	Not Vaccinated		
**Type of event**				
Hospitalisation	19,104/40,102	33,950/75,843	1.06 (1.05–1.08)	0.87 (0.86–0.88)
Deaths COVID19	10,212/40,102	8198/75,843	2.36 (2.29–2.42)	1.04 (1.01–1.06)
ICU ^c^/Deaths COVID19 ^d^	10,366/38,620	9238/73,120	2.12 (2.07–2.18)	1.01 (0.99–1.04)

^a^ RR—Relative Risk; ^b^ CI—Confidence Interval; ^c^ ICU—Intensive Care Unit; ^d^ excluded Reggio Emilia (data on ICU not available).

## Data Availability

The data presented in this study are available on request from the author (M.M.).

## Supplementary Materials

The following are available online at https://www.mdpi.com/article/10.3390/vaccines9070716/s1, Table S1: The RECORD statement—checklist, Table S2: Coding algorithms to identify comorbidities and drugs.
